# Dimensional Taxonomy of Data Visualization: A Proposal From Communication Sciences Tackling Complexity

**DOI:** 10.3389/frma.2021.643533

**Published:** 2021-04-19

**Authors:** Victor Cavaller

**Affiliations:** Department of Information and Communication Sciences, Universitat Oberta de Catalunya (UOC), Barcelona, Spain

**Keywords:** index terms: data visualization, dimensional taxonomy, communication process, communication theory, knowledge transfer, complexity

## Abstract

This article consists of a conceptual analysis—from the perspective of communication sciences—of the relevant aspects that should be considered during operational steps in data visualization. The analysis is performed taking as a reference the components that integrate the communication framework theory—the message, the form, the encoder, the context, the channel, and the decoder—which correspond to six elements in the context of data visualization: content, graphic representation, encoding setup, graphic design and approach, media, and user. The study is focused accordingly on the dimensions that these elements describe: the degrees of abstraction of the information, the functionalities of the tool for the graphical representation, the specifications for the setup of the visualization, the approach modes to the context by the graphic design, the levels of communication efficiency in the media, and the requirements of the visualization perceived as values from the user experience side. The unfolding of these dimensions is undertaken following a common pattern of six organizational layers of complexity—basic, extended, synthetic, dynamic, interactive, and integrative—according to the analytical criteria. The results of the detailed study, based on an extensive scientific literature review, allow the design of a dimensional taxonomy of data visualization built on a matrix structure where these elements act as factors of completeness and the layers act as factors of complexity. As a conclusion, an object-centered model constituted by an ordered series of phases and achievements is proposed as a guide to complete a systematic process of data visualization.

## Introduction

### Complexity as a Challenging Parameter to Integrate in Data Visualization

Over the past decades, visualization and complexity have received extensive scientific attention, and there has been a huge increase in the number of publications dealing directly or indirectly with their relation. Emergent complexity in systems theory is described as the distinctive novel properties or behaviors that arise in organizations from the interaction among their components (Gibb et al., 2019). Adding complexity is the common response of organizations under the influence of controllable and uncontrollable factors, by means of which they adapt themselves to changes in the environment. In complex systems, emergent properties are provided by networks of internal processes and hyper-processes in order to accomplish a particular function, which means that there is a scale factor involved in their structure. Complexity is deeply embedded in organizational dynamics, and it has become a real challenge for data visualization. If complexity characterizes in general any organization or phenomenon, by extension, the methods and techniques to visualize them must be accordingly modified or eventually adapted to capture the dimensional structure and scaled dynamics that configure the object.

Among the fields in which publications about complexity have reached more popularity in the last few years, modeling of biological ecosystems (May, 2019), social complexity (DeLanda, 2019), self-organization in statistical mechanics (Wolfram, 2018), ecological complexity (Allen and Starr, 2017), and economic complexity (Hausmann et al., 2014) can be highlighted. In a similar review focused on visualization, publications in cartography (Kraak and Ormeling, 2020), perception and design (Ware, 2019), sequencing technologies (Katoh et al., 2019), molecular visualization and analysis (Goddar et al., 2018), flow visualization (Yang, 2018), multivariate density estimation (Scott, 2015), neural networks (Yosinski et al., 2015), genetics (Hu et al., 2014), information and knowledge (Börner, 2015), and system visualization, analysis, and design (Munzner, 2014) have obtained an overwhelming impact.

In the particular field of data visualization, from its very beginning, pioneering works from authors such as Bertin (1967), Tufte (1983), Schneiderman (1996), Horn (1998), and Wilkinson (1999), followed by precursors such as Fayyad et al. (1996), Hoffman et al. (1997), Hoffman et al. (1999), Fayyad and Uthurusamy (2002), Manovich (2001, 2011, 2013, 2016), and Few (2011) up to the most prominent authors in the field such as Cairo (2013), Keim et al. (2013), Börner and Polley (2014), Heer and Agrawala (2006), Heer and Scheiderman (2012), Viegas and Wattenberg (2006), Wattenberg et al. (2016), or Benoit (2019) with many others, have made a great effort to ground data visualization on scientific principles.

### The Lack of an Integral Data Visualization Taxonomy to Tackle Complexity

Data visualization and complexity as scientific topics are undergoing a period of consolidation with an increasing and overwhelming number of scientific publications and specialists working on these fields. However, along with this positive impression, a more detailed overview suggests that linked problems remain unsolved:

1) From the object side, at any scientific discipline where the concept of complexity appears, it refers to objects constituted by interconnected layered networks; however, there is not a common proposal for a pattern of complexity in phenomena from both an organizational and analytical perspective.
2) From the subject side, the current limits dealing with the issue of complexity do not lie so much in its evidence or even characterization but rather have to be sought in the fact that the complexity of the object obliges the particular adaptation and sophistication of data visualization methods which call for a definition of their analytical potential to describe it.
3) Finally, from the practice side, data visualization is driven by highly demanding standards—its universal application as a tool, its specialization and versatility, and its need for effective and immediate results.

The root cause of the above-mentioned problems is the absence of an operative standard for the implementation of data visualization. As a consequence, the main deficit, repeatedly observed throughout this review, is that data visualization is still affected by a serious lack of systematicity which ultimately—from the perspective of communication sciences—can be summarized as the lack of an integral taxonomy.

There is no science without its own taxonomy. Taxonomy is the practice used by any science to clarify itself by classifying its concepts, being thus an exercise of self-explanation about its fundamentals. Data visualization occupies a central position as an applied science—in an intersection among statistics, semiotics, computer science, graphical design, and psychology, in close relation to communication sciences—which means that the meta-analysis required in order to generate a taxonomy must be performed over multiple scientific disciplines. Being central paradoxically represents a weakness.

Despite the fact that there have been tentative approaches to define a taxonomy in particular areas of data visualization (Schneiderman, 1996; Heer and Shneiderman, 2012; Ruys, 2020), the critical requirement for an integral taxonomy is a pending workload, and it is currently having a negative impact on both its consolidation as a rigorous technical method and on its recognition as a scientific discipline, beyond its instrumental use. Faced with this situation, it is appropriate to shed light on the foundations of the discipline of data visualization—understood as a communication process—in order to provide a solid ground for its systematic application. To achieve such purpose, a key action is required. Complexity has to be integrated as an internal parameter in the configuration of its operative. As complexity is a factor that constitutes the object and conditions the subject, data visualization needs to undergo a conceptual analysis object-centered on organizational complexity, which in turn must be tracked to each of the components of the communication process that participates in data visualization. This article is focused on this objective.

### Communication Components and Layers of Complexity in the Data Visualization Process

Any scientific research inquiry follows three procedural stages when managing data: data formalization, data analysis, and data visualization, which, respectively, transform observations and measurements into data, data into information, and information into knowledge. Formal data appear as a result of preprocessing operations, information appears as a result of data analysis, and knowledge appears as a result of data visualization. Data visualization can be transversely used as a tool in both processes of data formalization and data analysis, but ultimately, it constitutes the final and synthetic visible stage where the results of data analysis are reported. In fact, by means of the accuracy of data visualization, the success of any data processing is evaluated. In order to provide instruments from communication sciences that can contribute to the process of transforming data into understandable information and information into valid knowledge, it is necessary to deal with data visualization in a systematic way covering the totality of the factors that are involved in its process.

The first step to start a thorough review of these factors is to identify the following elements that participate in data visualization understood as a communication process:

- the *content*, the data, and information to be communicated
- the *graphic representation* of this content
- the *encoding* of the information integrating data and graph specifications
- the *design* adapted to the *context*, the audience, or the target
- the *media* by which the visualization is published and disseminated
- the *user* who receives the visualization

The proposal of these elements is not arbitrary. “Data visualization uses principles, concepts, techniques, and theories that come from multiple backgrounds: programming, web design, semiotic, or psychology” (Aparicio and Costa, 2014). However, from the point of view of the communication theory, these core elements are embedded in data visualization, beyond its background and application, in so far as they correspond to the most widely accepted framework of the communication model (Shannon and Weaver, 1949; Schram, 1954; Berlo, 1960; Rothwell, 2004; Barnlund, 2008). The elements are as follows:

1) MESSAGE: *what* things are communicated according to the context
2) FORM: *how*, in which form and by which *tool*, the content is communicated, taking into consideration the media through which it is broadcasted
3) SENDER: *by whom* and *why* the communication is provided and encoded in a singular setup observing the receiver
4) CONTEXT: *what* is the scenario *where* the communication takes place
5) CHANNEL: *through which* medium is the information communicated
6) RECEIVER: *to whom* the information is addressed

These six elements must be considered as factors of completeness in data visualization. The failure to observe any of them is a recurring cause of miscommunication and misunderstanding. Data visualization constitutes a process of communication, the efficiency of which is conditioned by the actions that these elements imply: the selection of the content, the formal representation of the information, the encoding and setup of the visualization, the graphical design appropriate to the context, the adaptation to the medium, and the observation of user preferences. Furthermore, understanding the completion of these actions as a critical success factor, they must be undertaken considering their interconnection which plays a critical role and can be expressed by means of the following practical questions:

1) What content does the sender want to communicate and to what *degree* of abstraction?
2) In which form? Which *functionalities* from which tools are appropriate for the graphical representation to be integrated in the pursued channel?
3) Once content and form are defined, what *specifications* must be applied to the setup of both data and graphical representation in order to adapt to each other?
4) What are the approach mode and the graphical design suitable to the context? What *properties* does the visualization have to meet depending on the target or audience?
5) What characteristics must the visualization contemplate in order to make it efficient according to the media where it is projected? What are the *levels* of communication efficiency that must be achieved?
6) What *requirements* must be observed from the user’s experience in order to improve understanding of the topic?

### Objectives and Method: Building a Taxonomy

The above questions highlight six *dimensions* of the communication process that, conditioning the systematic procedure of data visualization, must be accurately studied:

- the degrees of abstraction of the information
- the functionalities of the tool for the graphical representation
- the specifications for the setup of the visualization
- the approach modes to the context by an appropriate graphic design
- the levels of communication efficiency in the media
- the requirements of the visualization perceived as values from the user experience side

The definition of these dimensions leads to the equally important issue of internal order in which they must be unfolded. From previous studies about data analytical procedure (Cavaller, 2008; Cavaller, 2007), it has been shown that, as a general rule, the construction of indicators applied to data analysis is correlated with the layers of organizational complexity that exist in any organized entity or phenomenon:

1) Basic layer: basic interactions
2) Extended layer: multivariate relationships
3) Dynamic layer: distributions or multi-relational dynamic
4) Synthetic layer: internal logics or processes
5) Interactive layer: system as architecture of hyper-processes
6) Integrative layer: organization as ecosystem

Given that the layers of complexity of any object or phenomenon condition the structure of the analytical procedure, data analysis imposes a scale approach on data visualization in an object-centered way. Consequently, the sequential and detailed unfolding of data visualization—covering degrees, functionalities, specifications, modes and properties, levels, and requirements—must be internally described through cross-cutting layers.

Taking this conception as a starting point of the review and the analysis, the goal of this article was to design an *object-centered data visualization model*, organized in two axes:

- as a set of gradual approaches to the complexity of the dimension that is managed
- by means of the progressive completion of the corresponding communication component

As a result, a *dimensional taxonomy* of data visualization based on a matrix structure—where the elements that participate in data visualization act as factors of completeness, and their development in layered dimensions act as factors of complexity—is proposed (see Table 1).

**TABLE 1:** Matrix architecture of factors of completeness and complexity for the design of the dimensional taxonomy of data visualization according to the components of the communication framework theory.

			Communication framework component					
		Elements	Message	Form	Encoder	Context	Channel	Decoder
		Categories	What	How	By whom &why	Where and when	Through which	To whom
			**Factors of completeness**					
			**1**	**2**	**3**	**4**	**5**	**6**
		**Elements**	**Content**	**Graphic Representation**	**Encoding Set-up**	**Graphic Design and approach**	**Media**	**User**
		**Dimensions**	**Degrees** of abstraction	**Functionalities** of the tools for the graphical representation	**Specifications** of the set-up of the visualization	Approach **modes** and **properties** of visualization	**Levels** of communication efficiency	**Requirements** from the user experience side
		**Layers**	**Questions for the dimensional analysis**					
**Factors of complexity**	1	**Basic**	What content do you want to communicate and to what degree of abstraction?	In which form? Which functionalities from which tools are appropriate for the graphical representation that is pursued?	What specifications must be applied to the setup of both data and graphical representation in order to adapt each other?	What are the approach modes and the graphical design suitable to the context, target or audience?	What characteristics must be contemplated to achieve the levels of communication efficiency according to the media?	What requirements must be observed from the user's experience in order to improve understanding of the topic?
	2	**Extended**						
	3	**Dynamic**						
	4	**Synthetic**						
	5	**Interactive**						
	6	**Integrative**						

It must be observed that building the proposed taxonomy, the *theoretical framework* of communication sciences is projected as the *practical framework* for the dimensional analysis of data visualization. Meaning that in order to validate it, this article has been focused on an extensive systematic review of the scientific literature and on a conceptual analysis about the relevant aspects that have been considered both in practice and in the current debates about data visualization, categorizing them into topical groups taking as a reference those components and layers.

## Content and Degrees of Abstraction of Information

The first node of the communication framework is the message or the content of the communication. The first of the main functions of data visualization is to communicate a message: generally, information about an event, a phenomenon, a process, a system, or in general, any observable subset of the real world. At this starting stage, the assumption of the quality of data about the object is accepted as a fact because it should result from previous tasks of data formalization and analysis. Data visualization, from the perspective of the content to be represented, must distinguish six degrees of abstraction of information which correspond to six layers of organizational complexity.

### Parameters, Sample, and Descriptive Statistics

In practical terms, data visualization can be faced with three potential initial scenarios: a requirement of data visualization without previous data formalization, without previous data analysis, or, in the best case, with both data formalization and analysis previously performed. In the first scenario—that could be called agile, *adhoc*, or express demand—data visualization procedure must introduce a delay to examine the target in detail, to seek evidence, and to detect the different properties which presumably can be sustained by available data, in order to complete a proper answer to the requirement. The so-called data wrangling or data preprocessing operations are required before data analysis; such operations include data cleaning, matching, organization, and aggregation (Chen et al., 2015). In the second scenario, once a formalized dataset has been obtained or is available from a system of information, the actions to be carried out can directly jump to check whether the target can be delimited and whether a reduced and representative sample for a deeper analysis is available. In the third scenario, as the attention has already been focused on the particular issue, the consequent step is to select the data and constitutive relations that adequately answer the visualization requirement. In any case, evidence must exist and must be reducible to parameters and measurable. The *congruence* as the essential quality of being in agreement with the real-observed facts should be the principal and basic characteristic of data visualization.

### Clustering of Parameters: The Construction of Indicators as Evidenced Relations

A second degree of abstraction of information is reached when the requirement for data visualization needs adding and accumulating new observed properties about the subject to the focus. Different dimensions, traits, or aspects about the same reality are defined by different parameters as variables in a way that aggregating them by mathematical calculations leads to the construction of indicators that make their relations visible which “you otherwise would have been blind to if you looked only at the naked source” (Yau, 2013). The process of aggregating variables describing parametrical relations needs a thorough investigation, comprehensive in scope. A formal condition of this clustering can be defined as *exhaustivity*, the need to address all aspects without omission.

### Multi-Relational Dynamics: Set of Variables’ Distribution

The next degree of abstraction of the information is focused on the dynamics which refers to the multiple and observable distributions and relationships between sets of variables. It is understood that prior to data visualization, data analysis has been carried out in terms of detecting correlation or causality between variables. The definition of the relationships, as patterns in the dynamics, between sets of variables is considered as explanation of the variations observed in the phenomenon. A pattern is defined as any regularly repeated arrangement or relation in or between a set of parameters that modifies others or changes itself according to its distribution. Among all reasonable explanations, the best one covers the greatest spectrum of observed relationships or fits well enough to a sufficient portion of all the available information. The *consistency* is the modal quality—of being in the harmony, compatibility, and uniformity—that the explanation with the observation of particular distributions should pursue when dealing with the content of data visualization.

### Conceptual Synthesis and Symbolic Abstraction of a Process

In case of wanting to visualize a complex phenomenon, usually associated to a process, the definition of the parameters, the construction of indicators, or the detection of interconnected factors or patterns is not enough because the abstraction required is an, more than probable, explanation. Explaining a phenomenon as a set of separated dynamics is not sufficient either. The fourth degree of information abstraction involves the conceptualization of the internal relationship, the sequential process, and the vector direction that describes a phenomenon or lies behind the events. The nature of the interconnection between the dimensions of a process has to be observed as an objective condition of having a *logical unity in coherence*. When an explanatory model is involved as a communication message, data visualization requires a previous conceptualization, summarizing the accepted premises about the object logically interconnected.

### Layered Processes, Hyper-Processes, and Systems: Experimentation and Testing Hypothesis

When considering systems where hyper-processes—resulting from the coexistence of interconnected processes—are involved, a higher degree of abstraction in information must be achieved. The internal complexity of a phenomenon needs the definition of the layers where each constituent process takes place. The object of data visualization at this level goes from what was initially perceived as an isolated process to its interaction with other processes that condition each other, defining a network of system functions and their interactions.

Figure 1 shows the graphical representation of data on the layers of parallel activities undertaken by a university, illustrating how they participate in scientific research and technological development. The multilayered structure describing a hyper-process model is a clear expression of the crucial ability of systems to adapt to the complexity of the changing environment.

**FIGURE 1:**
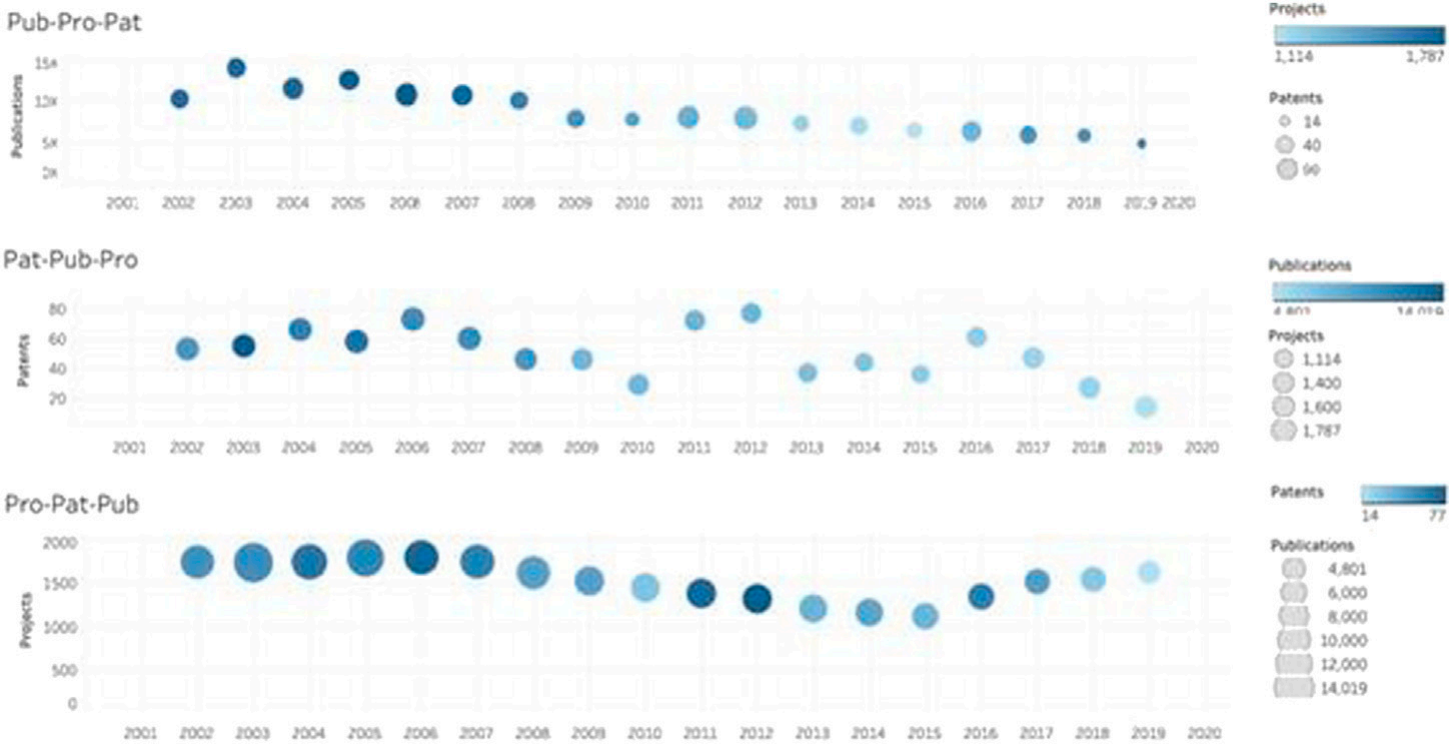
Detail of the interactive diagram map of the evolution of projects, patents and publications, considered as layers of parallel outcome processes of UPC in the period 2002–2019. Source: Cavaller et al. (2020), Own elaboration based on Future UPC data with Tableau.

Scientific progress implies the proposal of competing explanatory models, the certainty of which cannot be achieved. So there being no *verifiability* but *falsifiability* by experimentation (Popper, 1959), the evaluation of the confirmatory or falsifying value of evidence about a hypothesis depends on their demonstrative condition, which data visualization must facilitate in order to achieve scientific consensus.

### Abstraction in Scientific Modeling as a Reconstruction of an Organization

The degree of abstraction of the information is correlated with the complexity of the entity from which data have been obtained and data visualization has to show. The procedure of grouping a network of interactive processes in different layers is definitely dealing with the highest level of complexity that culminates the scope of data visualization in which an organization within its environment is explained. Scientific *modeling* and simulation are the results of a simplification and abstraction of human perception and conceptualization of reality that in turn come from physical and cognitive constraints. Modeling allows scientists to implement their *reconstruction*, simulating the program or code of the organization, future behaviors, visualizing scenarios, manipulating, and gaining intuition about the entities, phenomena, or processes being represented, for managerial or technical decision-making. At this level, uncertainty is a transcendent condition characterized by limited knowledge which ranges just beyond the experimentation in order to achieve a holistic view of a phenomenon.

## Graphical Representation and Tool Functionalities

Visualization has been defined as “a transformation of quantified data which is not visual into a visual representation”—“a remapping from other codes to a visual code” (Manovich, 2001; Manovich, 2011). Once the answer to the ominous question—which data in which degree of abstraction related to which level of organizational complexity about which object is required to be represented—is clear, the next question is: What is the ideal graphic representation to visually transform these data with a strictly functional orientation? This decision is not trivial. Principles of graphic communication, studied by semiology or semiotics, under which diagrams, networks, and maps or any sign in general are used, have been designed for the production of meaning in their close relation to the analysis of the information that they represent (Bertin, 1967). Here, it is worth remembering that one of the most recurrent errors in data visualization is to confuse the criteria for the selection of a proper graphic representation of data with the criteria of the graphic design of the visualization. The graphic representation from a functional point of view is directly related to the nature of the content to be displayed. In this sense, six different *object-oriented* graphic representations with six different functionalities can be defined.

### Basic Functionalities for a Descriptive Graphical Representation

The basic functionalities in the graphical representation of data are associated with a descriptive visualization of the parameters that depict a phenomenon. Information visualization relies on two key principles: reduction by the use of graphical primitives such as points, straight lines, curves, and spatial variables such as position, size, shape, or movement “to represent key differences in the data and reveal patterns and relations,” privileging them over other visual dimensions (Manovich, 2011). The development and formalization of statistical tools for the analysis and graphical representation of data have had a great impact in the field of visualization (Friendly, 2006). Graphs are useful to show relationships among variables—how a whole is divided into different parts, how variables have changed over time and their range, when and how data are connected, what are the trends, and how changes in one variable affect another—or to obtain a sequence in the development and transformation of trends or patterns. The main quality that is required from a graphic representation is to be descriptive. In this sense, the *evidentiality*, the condition to provide evidence, in an illustrative, expressive, and depictive way, is an essential condition by means of which the quality of the graphical representation in data visualization is evaluated.

### Advanced Functionalities for a Relational Graphical Representation

Multivariate or relational visualization involves the observation of multiple measurements and their relationship. There are different methods of visualizing a multidimensional or multivariate reality capable of covering a wide spectrum of inputs and outputs, associated with different analysis techniques and methodologies. “Data can be aggregated in many ways before being visualized in charts, profoundly affecting what a chart conveys” (Kim et al., 2019). In general, the need to express comparison, correlation, distribution, proportions, and hierarchy relationships in a dataset requires advanced functionalities in the visualization design. Two of the main principles of graphical integrity defined by Edward Tufte are referred to as *proportionality* and disambiguitty. “The representation of numbers, as physically measured on the surface of the graphic itself, should be directly proportional to the numerical quantities represented. Clear, detailed, and thorough labeling should be used to defeat graphical distortion and ambiguity” (Tufte, 1983). The property that pursues advanced forms of graphic representation is *integrity*, the formal condition of maintaining a direct proportion in the scale relationship of the parts with the whole and with the unit of measurement, without distorting the degree of interdependence of the variables.

### Functionalities for a Graphical Representation in a Dynamic Visualization

Dynamic or multi-relational visualization represents a reality where all the factors—defined as set of related parameters—are interconnected, and therefore there is interdependence between them, and consequently, their network position changes according to a spatial or temporal joint distribution. “Data visualization is an efficient means to represent distributions and structures of datasets and reveal hidden patterns in the data” (Chen et al., 2015). A dynamic visualization of data has to be facilitated by functionalities of tools for the transmission and understanding of the global and interconnected networked nature of a reality that is itself dynamic. Modeling of dynamic interaction networks has traditionally been supported by graph stream techniques or dynamic graph models (Harary, 1969). Among multiple applications, data visualization is useful for social learning analytics providing “additional information about actors and their behaviors for decision-making in online distance learning” (Hernández-Garcia et al., 2015) The *schematicity*, the ability to present in a schematic way the main features of the connectivity of the phenomena, allows the evaluation of the dynamic graph quality.

### Process Graph, Info Graphics, and Motion Graphics: Representing Processes

**FIGURE 2:**
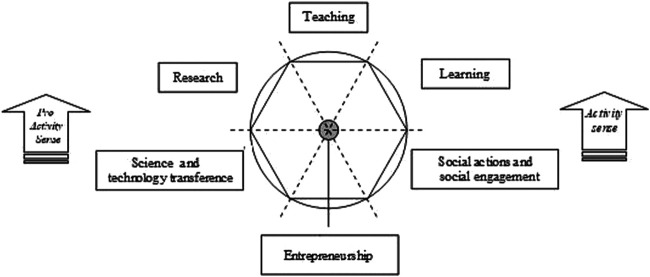
The Sextuple Helix Model of the KT sequential and cyclical activities in higher education (Cavaller, 2020).

Process visualization must describe the internal logics that lie behind phenomena. Once the interdependence relationships between the different factors or dimensions of a phenomenon are known, the existence of its internal logic can be inferred, and therefore it is possible to define an explanatory model and proceed to its visualization. However, in order to obtain a synthetic visualization that brings together the different dynamic perspectives of the same reality, continuing with a quantitative gradation in the abstraction of information and with its corresponding visualization is meaningless or clearly insufficient. The natural path to parameterization and visualization requires a qualitative leap that is made through symbolic abstraction with the use of info graphic representation and animation techniques (Harrison et al., 2015). The graphic representation at this level of complexity is done through process graphs, graph processing workflows, info graphics, and motion graphics (Curcin et al., 2010; Riazi and Norris, 2016; Microsoft Visio, 2020), or by means of diagrams, maps, or info graphics, it can display chronological, comparative, flow diagrams, anatomical, statistical, geographical, hierarchical or hybrid forms (Cairo, 2013; Manovich, 2016; Inequaligram, 2020). The quality of the process of graphical representation is defined by its objective condition of expressing the logic of their transformation flow, which refers to its *sequential* or *flow logicality*. In Figure 2, a process graph describing the Sextuple Helix Model for the assessment of universities based on KT processes is shown. The activities—as nodes—are proposed, in a two-way cyclical sequence, for their correspondent accounting and mission values (Cavaller, 2020).

### Interactive Graphics: Hyper-Process and System Graphical Representation

What is the ideal form of graphical representation of a phenomenon when its internal logic is also changing, complex or/and simultaneous processes in different layers at different scales are interrelated? Hyper-process or system graphical representation is needed to observe the constituent layers when describing the architecture of the systems where different processes coexist. The graphic representation of the data at this level must allow the user to interact with the visualization in order to know independently or together the different layers that are integrated into the phenomenon and their connection. One of the most common forms of graphic representation for interactive visualization is the interactive map. In general, interactive graphics are a type of graphic representation, which points to a demonstrative condition of an explanatory model, the ability to show evidence that verifies or refutes a hypothesis or theory that is defended. This property of *complex evidentiality* referred to a graphical representation means that its quality is evaluated by a demonstrative condition, the ability to give detailed, interactive, and *ad hoc* access to evidence of complexity, in order to demonstrate all the factors of a theory that is defended.

### Convergence of the Symbolic and Analytical Path: Integration of Scientific Data Visualization

Multidimensional phenomena structured in different layers of processes, where different organizational systems are involved, make their graphic representation an extremely complex matter. This difficulty has led to the need to develop new functionalities in the visualization tools that allow a comprehensive and holistic representation. At this level of complexity, the convergence between the symbolic and the analytical paths in data visualization prevails and requires that the graphic representation of data be accompanied in turn by a figurative or symbolic visual reconstruction of the reality of the phenomenon. This requirement is easily observable in scientific visualization, such as in modeling projects of biological systems (Ambicon, 2016) or in the field of medicine (Jang et al., 2014), to name a few examples, thanks to the extensive use of computer aided systems (SystemsBiology, 2016). The quality of a scientific visualization is evaluated by the capacity of reconstitution of reality in its entirety, even in what is not known in detail. The graphic representation associated with scientific modeling and simulation is characterized by the ability to represent the *intricacy* of phenomena internally and in their relationship with their environment, reconstructing those elements in which no sufficient evidence is available and posing them for a future demonstration.

## Encoding Specifications and Configuration Settings

The third node of the communication framework is the encoder, and its action is the encoding or the communication configuration. The main function of data visualization associated with this node is to communicate, so as to add meaning to the data and transform it into information. Visualization is the discipline that consists of “transforming data into meaningful information” (Benoît, 2019), and this transformation is made by encoding.

### Data Formalization *Adhoc*. Setting up Data and Plotting Elements for Descriptive Visualization

The first step in the basic configuration of data visualization is to specify and verify basic elements selected such as parameters, constants and variables, scale, data range, sample, legend, and labels, and to check them in preliminary views in order to manipulate and ensure the accuracy of the representation. The basic operations to be performed in this phase have been proposed—in a synthetic way—as tasks grouped into three high-level categories: 1) specification of data and views (visualize, filter, order, and derive), 2) view manipulation (select, navigate, coordinate, and organize), and 3) process of analysis and provenance (record, note, share, and guide) (Heer and Shneiderman, 2012). Visual analysis tools, such as Profiler, have been designed for assessing quality issues in tabular data such as missing, erroneous, extreme, and duplicate values that undermine analysis and are time-consuming, applying “data mining methods to automatically flag problematic data” and suggesting “coordinated summary visualizations for assessing the data in context” (Kandel et al., 2012). The specifications for a basic encoding of the data and its graphical representation pursue *accuracy*, an essential quality of being correct or precise for a basic visualization.

### Multidimensional Transformation

The configuration for the visualization of a multivariate set is basically solved in its transformation to a data matrix with rows and columns, representing cases and variables. There are different theoretical approaches or models that describe the procedural stages of configuring data visualization. “Card’s early model lists four successive steps: 1) the processing of raw data, 2) the transformation of data tables, 3) the mapping of visual structures, and 4) the transformation of the visual results (e.g., zooming and overview)” (Vande Moere and Purchase, 2011). Multidimensional transformation is related to the concept of visual metaphors and to the capacity for interaction (Kosara et al., 2006). The specifications for the configuration of multidimensional data and its graphical visualization pursue the preservation and detailed rigor of the proportions in the relationships detected between variables (Blackwell, 2011; GIS, 2020). In multidimensional data representation, such as 3D scatterplots, this is done by using a software volume renderer for display, combining it with InfoVis interaction methods such as linking and brushing (Kosara et al., 2004), selecting or displaying subsets of data and defining the relationship between them. In the Card’s model, novel visual metaphors represent the structure of, and the relationships within, complex data (Card et al., 1999; Vande Moere and Purchase, 2011). The property or quality that is pursued in a multidimensional configuration of data visualization is *multidirectionality*, a formal condition, which is defined as the ability to show the widest possible range of interrelationships between set of variables.

### Configuration of Data and Representation of Dynamic Multidimensional Distribution in Data Visualization: Integration of Applications

The next step in the process of configuring data visualization focuses on the dynamic relationships and distributions between groups of variables, combining and communicating different visualization techniques and methodologies, which generates a fundamental requirement for dynamic, compatible, and interconnected tools for visual encoding. “Tools do not exist in isolation, but within an ecosystem of related components” (Bostock et al., 2011). New tools have been designed to facilitate application integration in visualization design. However, “despite a diversity of software architectures supporting information visualization, it is often difficult to identify, evaluate, and reapply the design solutions implemented within such frameworks” (Heer and Agrawala, 2006). One successful example of this capability is dashboards, very useful embedded tools that allow the programmer to develop visualizations of known variables, dimensions, and relationships from a dataset (Klipfolio, 2020). Dashboards combine “multiple conventional data visualization styles to most efficiently and accurately be able to understand data,” “facilitating exploratory analysis and answering a multitude of new questions” (McCann, 2020). The difficulties in detecting and making the behavior patterns of dynamic distribution visible are associated with the difficulties of integration of the visualization tools (Heer and Agrawala, 2006). The modal quality that is required from the configuration of a dynamic visualization is its *versatility* in terms of interconnectivity and compatibility with other tools.

### Configuring Data Process Visualization

Following consecutive levels of complexity, the configuration is directed to the design and programming of algorithms that simulate the operation of the logical structure of the process that underlies the phenomenon to be represented graphically. The modeling of a process, in order to visualize it, includes different operational moments: 1) defining the flow diagrams and the forms of representation, 2) selecting inputs and outputs of the processes for each of the events and activities, and 3) obtaining or designing the algorithms that synthetically define their relationship in the analyzed process. The configuration has to point to the definition of an explanatory model that is represented and, therefore, to the logical structure that underlies (Curcin et al., 2010). An example of a software tool that allows the configuration of process visualizations by generating algorithmic art is processing (Reas and Fry, 2007; Terzidis, 2009; Greenberg et al., 2013; Processing, 2020). In machine industry and manufacturing methods, control systems such as the Supervisory control and data acquisition (SCADA) incorporate graphical user interface (GUI) and allow users to interact with electronic devices, computers, networked data communications through graphical icons, and audio indicator (Boyer, 2010; Siemens, 2020). The property that is sought in a configuration of the visualization of a process is being *self-explanatory*, an objective condition of being able to express the autonomous mechanics of a process easily understood.

### Specifications for the Configuration of the Data and Its Graphical Representation in an Interactive Visualization

Getting into the internal complexity of phenomena involves defining the different layers of sub- or super-processes that participate or overlap in strata, which in turn requires developing and mastering complex visualization tools. The specifications for the configuration of an interactive visualization are framed in the experimental and demonstrative stages of the research. Tools such as Vega-Lite—“a high-level grammar of interactive graphics”—allow the generation of visualizations to support analysis, supporting “data transformations such as aggregation, binning, filtering, sorting, and visual transformations including stacking and faceting,” composing specifications “into layered and multi-view displays, and made interactive with selections” (Vega-Lite, 2020). “Users specify interactive semantics by composing selection” abstractions that define “input event processing, points of interest, and a predicate function for inclusion testing. Selections parameterize visual encodings by serving as input data, defining scale extents, or by driving conditional logic” (Satyanarayan et al., 2017). “When building visualizations, designers often employ multiple tools simultaneously (Bostock et al., 2011), combining powerful visualization components (d3js, 2020) “to visually and interactively investigate transactional flow.” The property that is pursued in an interactive visualization is the *multidimensional operability* and the *transparency*, the ability to show the internal complexity and to manage data autonomously.

### Display Settings for Visual Reconstruction

In the comprehensive visual reconstruction of an organization, the convergence of data visualization and data analysis has become indispensable. The goal is to provide—in an interactive way—simultaneous calculation and visualization of the interconnected relationships among variables, distributions, and flow of processes in the different layers and phases of systems in organizations. “A useful starting point for designing advanced graphical user interfaces” is the Visual Information-Seeking Mantra of “seven tasks: overview, zoom, filter, details-on-demand, relate, history, and extracts (Schneiderman, 1996)” and to incorporate “the critical tasks that enable iterative visual analysis, including visualization creation, interactive querying, multiviewed coordination, history, and collaboration (Heer and Shneiderman, 2012). Visual analysis, modeling, and simulation of ecosystems and organizations are quite common, especially in the field of topological data analysis (Xu et al., 2017).

Complex adaptive systems modeling can be found in a wide range of areas from life sciences to networks and environments (CASModeling, 2020). Analysis and visualization of large networks can be performed with program packages, such as Pajek (Mrvar and Batagelj, 2016). The property that the configuration of an integrative visualization has to pursue is the *ubiquity* in order to accomplish a synthetic and holistic vision and analysis, which can be characterized as the capacity of understanding the complexity of a system by making it visible. The final step in the encoding of data visualization reaches the definition of the cross-layers of the functional system, which means to visually configure the vertical interconnection between the processes at their different layers.

**FIGURE 3:**
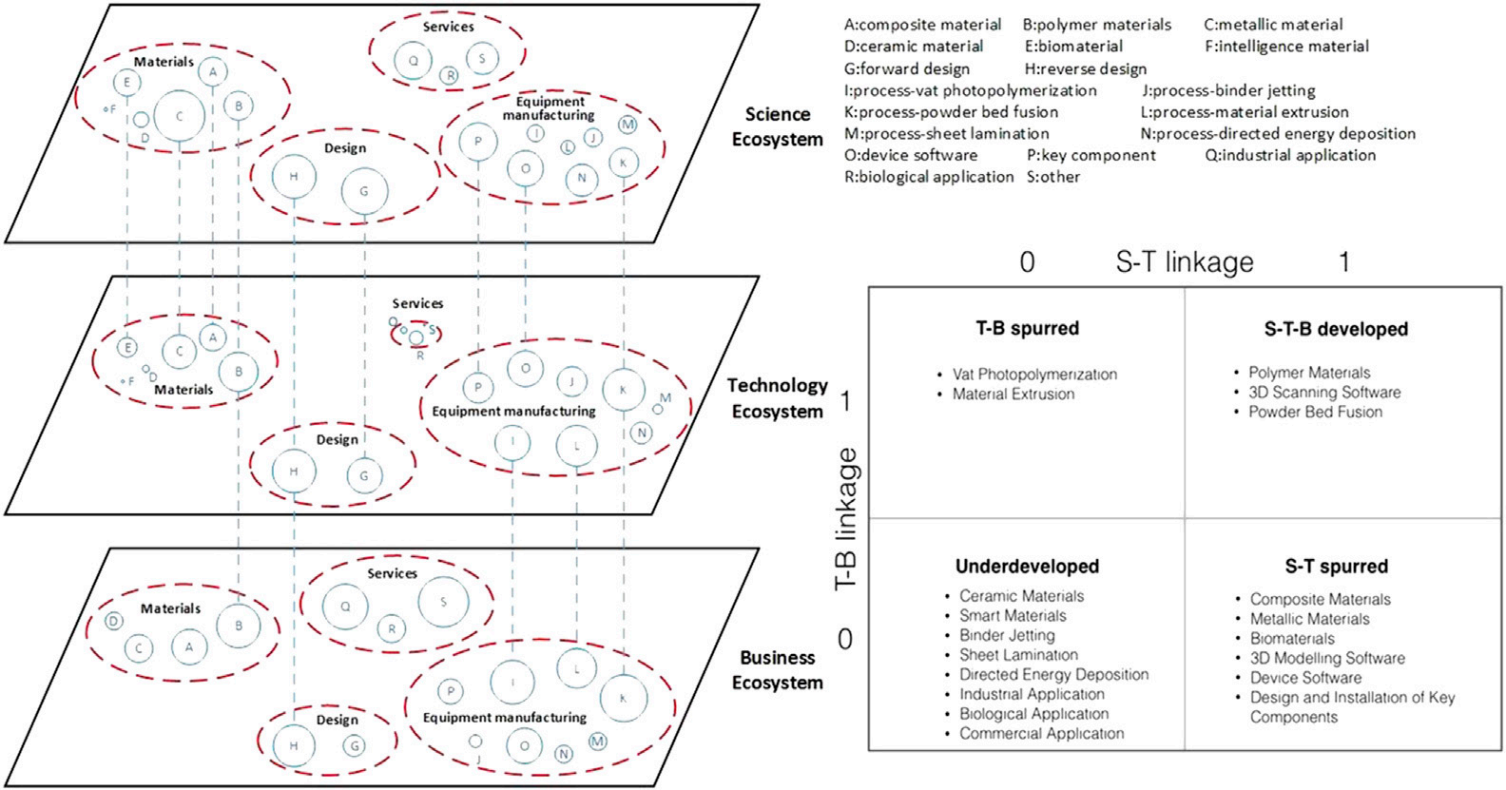
Example of cross-layer analysis and visualization of a collaborative network in a science–technology–business ecosystem. Source: Xu et al. (2017).

Figure 3 shows a representation of the multilayered innovation ecosystem that involves science, technology, and business sub-ecosystems as an example of cross-layer analysis of collaborative network to investigate innovation capacities (Xu et al., 2017).

## Graphic Design and Context: Modal Approaches and Properties of Good Data Visualization

The fourth node of the communication framework is the context, which in data visualization is developed by graphic design. Data visualization has to be adapted to the target and to the context where it is carried out through a suitable graphic design that captures the user’s attention “to convey ideas effectively, both esthetic form and functionality” (Friedman, 2008). The effectiveness of the design of data visualization is evaluated by its impact on the user, and it is explained by the mechanisms of human perception of esthetic forms in particular contexts. “The most effective visualizations capitalize on the human facility for processing visual information, thereby improving comprehension, memory, and inference. Such visualizations help analysts quickly find patterns lurking within large datasets and help audiences quickly understand complex ideas” (Agrawala et al., 2011). The context is the criterion that classifies the approach modes to visualization and the esthetic forms of graphic design adopted.

### Subjective Approach

Visualization must be meaningful. It has to pursue the properties of any communication act—clarity, concreteness, saving time, stimulating imagination and reflection, empowering the user, *etc*. For this purpose, from the perspective of computer-supported cooperative work (CSCW) studies, the idea of context and common ground being associated is important (Viégas and Wattenberg, 2006). In the subjective approach, the idea of context in its association with graphic design has to be defined considering the human–computer interaction (HCI). The principles of visual representation for screen design and the basic elements or resources used such as typography and text, maps and graphs, schematic drawings, pictures, node-and-link diagrams, icons and symbols, and visual metaphors should be observed. Engelhardt (2002) in his analysis of syntax and meaning in maps, charts, and diagrams establishes a classification of the correspondence systems between design uses and graphic resources (Blackwell, 2011). The principles of perception, visual processing, and the mechanisms and limitations of attention and memory, developed by the Gestalt School of Psychology—proximity, similarity, enclosure, closure, continuity and connection—define those mechanisms by which the human perception identifies patterns, forms, and organizations (Few, 2011), which explains that traditionally visualization reserves the spatial arrangement, the layout, for the most important dimensions of the data, to “code quantitative differences between objects and/or their relations” (Manovich, 2011). Complementing the coding that the brain automatically performs, the design can be used for recontextualization. The property that data visualization pursues through its graphic design in a subjective approach is *communicativity*, an essential condition or quality of being able to convey meanings from one entity or group to another through the use of mutually understood signs, symbols, and semiotic rules.

### Objective Approach

Data visualization plays a critical role in multiple professional and academic fields, which means that it needs to adapt to particular specifications. The objective approach points to the context of professional specialization; for that reason, the graphic design must be basically functional in nature. Communication focuses on how to identify, instantiate, and evaluate domain-specific design principles for creating more effective visualizations (Agrawala et al., 2011). Graphic design is associated with graphic representation that can help the audience to understand better the relevant information. For instance, contour plots, heat maps, scatter-line combo, 3D graphs, or histograms can be especially useful in meteorology and environment, whereas line graphs, bar graphs, pie charts, mosaic or Mekko charts, population pyramids, and spider charts are usually more useful in marketing. Graphic design, to be effective, has to adapt to the functional needs in such a way that it has to modulate other principles of visualization. For instance, in Harry Beck’s 1933 redesign of the London Underground station—because travelers only need to know the address and the remaining stops to reach their destination—aspects about the informational relevance of data were considered above others. From an objective approach perspective, the property that data visualization pursues through its graphic design is functional *adaptability*, a formal condition that refers to the ability to change in order to suit the needs of a new context or situation.

### Informative Approach: Design as a Semiotic Mode

When there is an informative purpose, the communication effectiveness of the graphic design—beyond the subjective and objective approach that is constricted by the deontological reporting principle “to keep to the facts”—is mainly conditioned by the need to attract the attention of the user. “In an era of narrowly focused media that is often tailored toward audiences with a particular point of view, data visualization—and data journalism in general—offers the tantalizing opportunity for storytelling that is above all driven by facts, not fanaticism” (Cohen, 2020). The properties that graphic design of data visualization must meet in an informative approach can be assumed as properties of journalism. “Besides images, design is coming into play as a crucial semiotic mode for making meaning. In news features, special reports, or data visualizations, we can find a rich and complex interplay of different semiotic modes, for example, text, image, and layout, which constitute the meaning-making process” (Weber and Rall, 2016). In the field of data journalism, numerous examples of application of data visualization can be found, which are used to help to tell a story to readers (Cohen, 2020). In a fast-changing informational environment, graphic design in data visualization fundamentally has to be dynamic (Weber and Rall, 2016). The property that data visualization pursues through its graphic design in an informative approach is the *appealingness*, a modal condition of showing attractiveness that captures or awakes someone’s interest.

### Commercial Approach and Persuasive Communication

In the commercial approach, the graphic designer does not only try to capture the attention and interest of the user but also tries to convince him of the benefits of a product and a service. Visual communication can be fundamental as a complement of social influence. “Skilled visual designers manipulate the perception, cognition, and communication intent of visualizations by carefully applying principles of good design” that “can be used to either emphasize important information or de-emphasize irrelevant details” (Agrawala et al., 2011). Graphic design at this level is oriented to the presentation of a service, a concept, or a product, in which a clear persuasive intention is implied. Color choice, use of shapes, page layout, composition and focal points, rule of thirds, golden mean or divine proportion, eye path or visual hierarchy, balance, movement, white space, pattern, repetition, structure, type styling, grids, and alignment and contrast are effective design principles of persuasive communication that can make or break your marketing’s effectiveness (Change Conversations, 2020). There are several types of graphic design that traditionally have been applied to marketing using “visual compositions to communicate ideas through typography, imagery, color, and form” such as visual brand identity, advertising, user interface, publication, packaging, environmental or way finding, art, and illustration (Cann, 2018). The property that data visualization pursues through its graphic design in a commercial approach is the *persuasivity*, an objective condition of being good at causing someone to do or believe something through reasoning or the use of temptation.

### Educational-Investigative Approach

In contexts where learning or research processes take place, the design of data visualization is a factor of great importance. The synthesis and summary of data must be given in clear, attractive, and comprehensive graphic visualizations that show the logic of the internal connection of the elements or factors that participate in highly complex phenomena.

**FIGURE 4:**
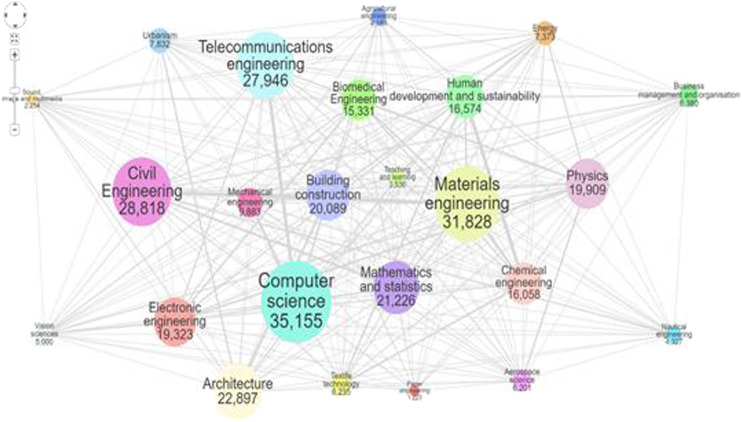
Areas of knowledge and their relationship based on the scientific production of UPC researchers. Source: Future UPC (2020).

On the other hand, visualization requires user interaction, so the design has to adapt to the different phases of the learning or research process, or of discovery, be demonstrative, suggestive, progressive, *etc*. In the process of designing interactive visualizations for learning process, where performance, trial, and error are fundamental parts, in order to attempt to balance expressiveness, efficiency and accessibility visualizations can be greatly enhanced by interaction and animation (Bostock et al., 2011). Educational and scientific research approaches usually pursue synthetic graphical designs adapted to technical profiles. Figure 4 shows “the UPC areas of knowledge and the relationship between them based on the scientific production of UPC researchers. Each node represents an area, and its size is determined by the number of activities in the portal FUTUR pertaining to it” (Future UPC, 2020).

Interactive visualizations are associated with techniques such as storytelling, which in turn are closely linked to graphic design. In her book “Design is Storytelling,” Ellen Lupton reflects about the maxim “design is problem-solving” and how “designers use simple forms to convey compact messages” (Lupton, 2017). Plot, emotional connection, and simplicity (Less is more) have been described as three storytelling techniques for graphic design (Schauer, 2017). Investigative journalism is also one of the most important sources for producing interactive data visualization designs. The latest editions of the Online Journalism Awards (OJAs, 2019) or the Data Journalism Awards (2019) provide numerous examples of projects that allow interactive exploration. In an educational-investigative approach, the property that a data visualization pursues through its graphic design is the *dialogicity*, a demonstrative condition that takes the dialogue as “an efficient motivational strategy in encouraging participation in common efforts” (Zimmermann, 2011) for knowing the internal complexity, the detail of a case, a story, or an event.

### Scientific Approach

The graphic design of data visualization in a scientific approach is a challenge that can be explained by different perspectives. From the point of view of collaborative experiences in applied research, it has been observed how “graphics are becoming increasingly important for scientists to effectively communicate their findings to broad audiences, but most researchers lack expertize in visual media” (Khoury et al., 2019). From the point of view of scientists, “figures have a prominent role in scientific publications and often take up the majority of time when preparing a manuscript. Scientists and engineers would greatly benefit from having the appropriate design knowledge to draw effective figures” (Cheng and Rolandi, 2015). From a technological point of view, there are a large number of programs that provide solutions to support research and scientific communication, such as CartoDB (Carto, 2020) or Vizzuality (cfse, 2020). Data visualization experts point out that “good visualization is a winding process that requires statistics and design knowledge” (Yau, 2013). In its application to scientific dissemination, “well-constructed graphics can widen the impact of research articles” (Marcus, 2010; Khoury et al., 2019). Some initiatives have been proposed in order to maintain “key connection between the sciences and the visual arts,” such as Design Help Desk, a project funded by the National Science Foundation that investigates the impact of visual design on scientific figures (Cheng and Rolandi, 2015). Finally, numerous companies in the field of visualization maintain a commitment to scientific dissemination and social responsibility associated with a vision that transcends the pragmatic use of visualization and data analysis (Periscopic, 2020). The property that data visualization pursues through its graphic design in a scientific approach is the *integrativity*, a condition of gathering in a visual unit the most detailed possible set of data and information of a complex reality with the possibility of interacting and experimenting with it.

## Media and Levels of Communication Efficiency

The fifth function of data visualization is to communicate relevant and objective information—understood as knowledge—in the most efficient way through the appropriate media. “The efficient communication of complex quantitative ideas” (Tufte, 1983) implicates the ability of being able to communicate successfully, minimizing the total resources of visualization taken in.

### Content Editing: Correctness, Completeness, Timeliness, Accuracy, Review, and Control

The communication efficiency in editing the content of data visualization is measured in relation to its correctness, completeness, timeliness, accuracy, form, purpose, proof, and control. Statements in the “Disclaimer” section of websites related to the care and the accuracy with which the online content is created together with the rejection of any responsibility in case of incomplete or incorrect information are common (Human abilities, 2020). This major concern indicates the substantive contribution of the quality of the media diffusion of information on data visualization evaluation. Systems that verify the quality of the information have become extremely important. Not respecting this fundamental principle can lead to problems of social perception. For example, it is known that in the field of web-based social data analysis, “tools that rely on public discussion, to produce hypotheses or explanations of patterns and trends in data, rarely yield high-quality results in practice” (Willett et al., 2012). The control of data quality and its visualization are subject of study, for instance, in the framework of experiences and projects such as ESS Visio (2020) of the European Commission, where “sharing visualization tools between National Statistical Institute” has been successfully proposed (Laevaert and Le Goff, 2020). The basic characteristic that data visualization pursues through its media edition for diffusion is quality based on content *rigor*, an essential condition associated to *reliability* and verifiability that includes other characteristics—mentioned above—such as correctness or completeness.

### Information Architecture and Navigability

Web navigability is a formal property that usually describes the ease with which a user moves through an “informational website”; therefore both the concept of navigability and the “guidelines for designing web navigation” for designing, managing, and augmenting effective link (Farkas and Farkas, 2000) applied to data visualization, and its diffusion in a communication channel, can be understood and assumed in similar terms. The *navigability* of data visualization can be conceptually examined along three dimensions: clarity of target, clarity of structure, and logic of structure (Wojdynski and Kalyanaraman, 2015). Effects such as reduction of search time, comprehension of content, and decrease of task time related to classical principles for the graphical design of interfaces such as “implications of memory, structure, and scent for information retrieval” (Larson and Czerwinski, 1998) must be equally considered in the dissemination of data visualization. Similarly, those contents related to the architecture of the information focused on the “structural design, organization, layout, and information” in the navigable space are applicable for data interactive visualization (SAP 2011; Uncharted Software, 2018; SAP 2020; Software, 2020). Associated with the concept of navigable space proposed by Lev Manovich (Manovich, 2001), the concept of mapping (Horn, 1998) has become one of the most prominent forms of visualization in the media associated with navigability as an exploratory activity—“Science mapping is a generic process of domain analysis and visualization” (Chen et al., 2015; Chen, 2017).

### Technology and Visual Tools: Accessibility

Communication efficiency of data visualization is conditioned by accessibility which refers to the modal condition of being “usable by people with the widest possible range of abilities” (Henry et al., 2014). Applying the basic principles of *accessibility* that have been described as recommendations in the framework of the Web Content Accessibility Guidelines (WCAG 2.0), data visualization must have content that is “perceivable, operable, understandable, and robust” (W3C, 2019). Perceptibility as an efficiency factor has to be qualified on a quantitative level, in light of the well-known Weber–Fechner psychophysical law, according to which “the perceived change in stimuli is proportional to the initial stimuli” (Fechner, 1966). Perceptibility leads to the requirement that “information and user interface components must be presentable to users in ways they can perceive” (W3C, 2019), taking into account the ranking visualization of the correlation between stimuli and perception (Friendly, 2009; Demiralp et al., 2014; Kay and Heer, 2015; Prakash, 2016). Operations on a visualization interface allow the identification of salient patterns at various levels of granularity (Chen et al., 2015) in order to “promote comparison of terms both within and across latent topics” (Chuang et al., 2012) for assessing the data in context (Kandel et al., 2012). In the data-driven era, the understandability of the user interface is crucial to make timely decisions (Keim et al., 2013; W3C, 2019), in areas such as social media (Bello-Orgaz et al., 2016) or geoinformatics (Zuo et al., 2016). The ability to be “interpreted and managed reliably by a wide variety of user agents, including assistive technologies,” which defines content robustness (W3C, 2019), has been observed in the field of “traffic data visualization” (Chen et al., 2015), social network (Hernández-Garcia et al., 2015), or designing animated transitions to convey aggregate operations (Kim et al., 2019).

### Mediality: Multimedia, Hypermediality, and Multi- or Cross-Platform

Communication efficiency of data visualization is related to the concept of *mediality* which is the ability to appear in a communication medium, conditioned to understanding audiences, which “is integral to creating and distributing media messages” (Grady, 2020). ‟Multimediality is the interconnection of various functions which can provide media (text, images, graphics, animations, simulations, and so on)” (Brdička, 2003). The principle of multimediality is “used to be perceived as a way of facilitating the application of a wide range of transmission media” (Klement and Walat, 2015). In the field of education and especially in e-learning, multimediality “has to be understood as a means to stimulate multiple sides to pupil`s perception” (Klement and Walat, 2015). Hypermediality refers to digital content that, in addition to being in multimedia format, is interconnected in its configuration in order to facilitate navigation by user interaction. Hypertextuality refers to hypermediality restricted to the web publishing format.

**FIGURE 5:**
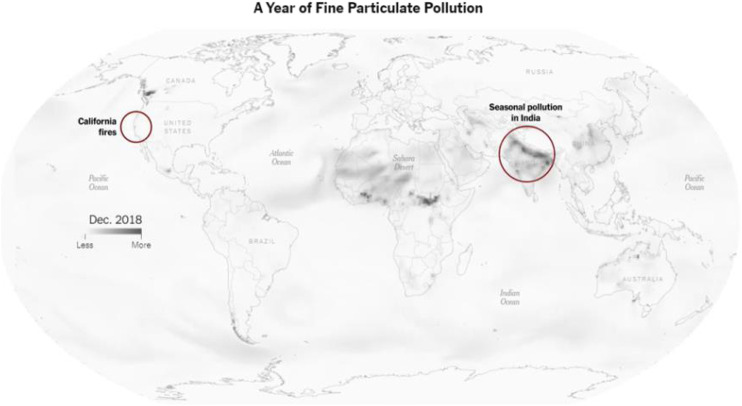
Screen capture of the Data Journalism Award (2019) Best visualization (large newsrooms). Winner: See How the World’s Most Polluted Air Compares with Your City’s. Organization: The New York Times. https://www.nytimes.com/interactive/2019/12/02/climate/air-pollution-compare-ar-ul.html.

Data visualization is closely related to multi-platform or cross-platform mediality forms “in the context of a wide range of distribution possibilities (e.g., online, mobile, and interactive games)” (Doyle, 2016). Multiples cross-platform data visualization solutions such as RGraph, Anychart, ZingChart, and DataGraph created by Visual Data Tools, such as Zoomcharts, are, among others, being developed by software companies. Criteria such as “avoiding repetition and increasing productivity” have been applied to assess cross-platform development approaches (Heitkötter et al., 2013). Figure 5 shows an example of how the results of scientific research can be integrated in data journalism through innovative visualizations including multimedia contents being potentiality broadcasted in multiplatform media.

### Media Interaction: Social Media, Cross-Mediality, and Data Storytelling

Communication efficiency of data visualization can be evaluated by its ability to assume different forms of interactive mediation between the user and the technology that gives access to the medium—“Interactive and dynamic graphics as part of multidimensional data analysis” (Cook and Swayne, 2007), “exploring high-dimensional data,” and providing “highly dynamic and interactive graphics” (GGobi, 2020). “Visualization framework for real-time decision-making in a multi-input multi-output (MIMO) system” has been designed using statistical inferences in order to provide “accurate visual measures/decision surfaces” (Ashok and Tesar, 2008). InSense, ManyEyes, and TweetPulse are some of the social big data applications that allow creating visualizations from collecting user experiences in collaborative environments through wearable data collection systems (Blum et al., 2006; Napalkova et al., 2018; WebLyzard, 2020). Data visualization is a key technical challenge when designing Cross Media games, employing “a wide variety of gaming interfaces based on stationary and mobile devices to facilitate different game experiences within a single game” (Lindt et al., 2005, Lindt et al., 2007) such as the alternate reality games (Walz, 2010). The evaluation of the efficiency of data visualization is also related to its capacity for transmediality, where consumers play an active role in different platforms and media (Chen et al., 2015). Investigative journalism also incorporates the concept of data storytelling or data narrative where ideas must be supported by data while maintaining and demonstrating rigor in their processing. Elements that participate in the narrative according to info graphic taxonomies have been categorized (Ruys, 2020). In the last decade, publications on the convergence of data visualization and data storytelling are experiencing rapid growth (Segel and Heer, 2010; Hullman et al., 2013; Kosara and Mackinlay, 2013; Knaffic, 2015; Lee et al., 2015). The *multimedia interactivity* or *participativity* is the ability to promote interactive access to users in order to spread a message or a story, a demonstrative condition that can be used to measure the communication efficiency of data visualization once it is projected in the media.

### Meta-Mediality: Augmented Reality, Hyperreality, and Mixed Reality

Once “life coaching has been presented as a collaborative social action of storing and sharing users life events in an open environment” (Bello-Orgaz et al., 2016), the next step is to enable visualization to recreate a reality from the media but especially beyond the media. “What happens to the idea of a ‘medium’ after previously media-specific tools have been simulated and extended in software? Is it still meaningful to talk about different mediums at all? Or do we now find ourselves in a new brave world of one single monomedium, or a metamedium” (Manovich, 2013, *borrowing Kay’s term*). Metamediality, applied to data visualization, can be understood as a transcendental condition in as much as its aim is to overcome the figure of the medium as intermediary, seeking to transcend the reality that it explains, creating a new one (Kay and Goldberg, 1977). Metamediality can be understood as a mix between metafiction and intermediality, ranging from augmented reality (AR) as an interactive experience, and hyperreality, where consciousness is unable to distinguish reality from a simulation, to mixed reality (MR) as the merging of real and virtual worlds to produce new environments and visualizations, where physical and digital objects coexist and interact in real time. The possibility of recreating and living the data visualization by the user constitutes a transcendental capacity of *experiment-ability* that defines data visualization when it transcends the medium where it is projected.

## User and Usability Requirements

In the visualization process and as a culmination of it, the requirements arising from the interaction and user experience must be considered, which are defined as components of usability. Usability is a “quality attribute that assesses how easy user interfaces are to use” and “also refers to methods for improving ease-of-use during the design process (Nielsen Norman Group, 2012). Some of these requirements are included in the ISO 9241-11, “The objective of designing and evaluating systems, products, and services for usability is to enable users to achieve goals effectively, efficiently and with satisfaction, taking account of the context of use” (ISO, 2020). These goals have been proposed as components of usability as parameters to be tested: learnability, efficiency, memorability, errors, and satisfaction” (Nielsen Norman Group, 2012).

### Perception: Learnability and Flexibility

The essential requirement that results from the user experience of data visualization is learnability, “how easy is it for users to accomplish basic tasks the first time they encounter the design? (Nielsen Norman Group, 2012) Learnability in data visualization can be defined as the attribution or basic quality necessary to enable a user to learn from it and learn to interact with it. To this end, “manual and automated chart specification to help analysts engage in both open-ended exploration and targeted question answering” have been developed (Wongsuphasawat et al., 2017) in order to facilitate the user experience: how people should use the information, what they should use it to accomplish” (Few, 2011). The *learnability* requirement leads to *flexibility*, the ease of operating interactively with all the possibilities with which the user and the system can exchange information: possibility of dialogue, multiplicity of ways to carry out the task, and similarity with previous tasks (Fernández Casado, 2018), ensuring user familiarity with visual analysis tools (Kim et al., 2019).

**FIGURE 6:**
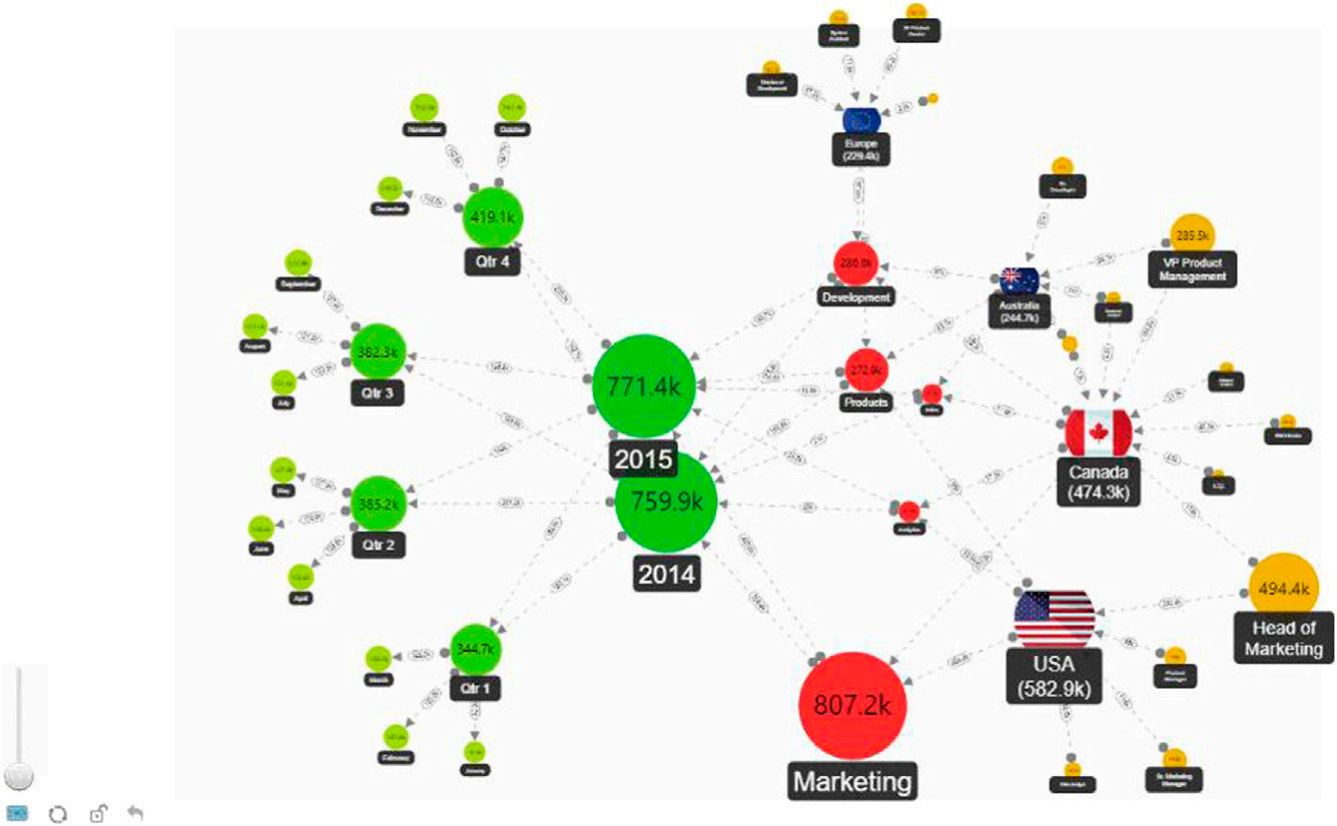
Screen capture of interactive ZoomCharts Network Chart Custom Visual for Microsoft Power BI which is supported on all platforms and can be implemented and customized by the user (ZoomChart, 2020).

The network chart shown in Figure 6 illustrates how innovative software and applications are leading a new open approach for data visualization that allows the user to customize the parameters of their preferences according to their own criteria.

### Accomplishment of Tasks and Efficiency

The second level of the user experience in the use of data visualization occurs when the user is active and has an autonomous experience. The consequent question formulated in terms of *efficiency* as a component of web usability is the following: “Once users have learned the design, how quickly can they perform tasks?” (Nielsen Norman Group, 2012). Here, efficiency is evaluated as a formal requirement of usability that is defined in terms of resources, such as time, human effort, costs, and materials (ISO, 2020) deployed for the accomplishment of tasks. Efficiency related to the accomplishment of tasks is a requirement that can be evaluated by observing the quality of the autonomous experience that the user has when using and interacting with the visualization. Efficiency in usability can be measured based on performance data–applying methods, similar to Ads Quality Score that is obtained by analyzing the relevance of the content, the loading speed, the quality, and relationship of the images, texts, links, *etc*. (Google, 2020). Obtaining major detail about the relation between user performance and experience is possible. For instance, in life-logging services, different factors of user experience are recorded. Sensors can capture continuous physiological data, such as “mood, arousal, and blood oxygen levels together with user activities” (Bello-Orgaz et al., 2016). The usability analysis can be supported by techniques of “exploratory analysis of dynamic behavior of individuals visiting a particular website” (Cadez et al., 2003).

### Performance and Effectiveness of Data Visualization in Knowledge Transfer

When the user operates with data visualization as an instrument for the representation, analysis, or visual communication of data the user’s actions are oriented toward how visualization facilitates the process of transferring information and knowledge so that efficiency becomes effectiveness, as a modal requirement of performance for the user that must be evaluated. The *effectiveness* as a measurable element can be defined as the “accuracy and completeness with which users achieve specified goals” (ISO, 2020). A classic example of communication effectiveness can be observed in the famous *Anscombe quartet* that Edward Tufte used to illustrate the importance of visualization as an instrument of analysis and therefore for the transfer of knowledge (Tufte, 1983). The effectiveness and *performance* as a component of the usability of a visualization comes from the use that visualization makes of the resources of the human visual system as a processor to detect patterns, trends, or anomalies, which explains the use of facilitating plugins based on perceptive factors. For example, in the field of “Designing Animated Transitions to Convey Aggregate Operations,” recent studies “indicate that staged animated transitions can improve the subject’s ability to correctly identify aggregation operations, although sometimes with longer response times than with static transitions (Kim et al., 2019).

### Proficiency and Memorability

A higher level of complexity in the requirements for good visualization based on the user experience is reached when the user is empowered by the acquired knowledge and expert mastery of the visualization tool. Here, the requirement that visualization must achieve is to enable the user to improve his experience by incorporating his own contributions or preferences, expanding the framework of action, and applying this experience to other cases. Here, it is necessary to consider the competencies of the user in relation to the configuration of the human brain, which in turn corresponds to the different dimensions of the human as a self-conscious being. When the user operates proactively, the spatial working memory (SWM) “plays an essential role in driving high-level cognitive abilities,” and it “is associated with global brain communication” (Liu et al., 2017). *Memorability* as a component of usability can be considered in terms of determining how fast and easy it is for the user to “reestablish proficiency” (Nielsen Norman Group, 2012) and has been developed in relation to visualization recognition and recall “what components of data visualization attract people’s attention, and what information is encoded into memory” (Borkin. et al., 2015). As in the case of effectiveness, reestablishing *proficiency* can be improved in an assisted manner. For example, “animation can help viewers track changes and stay oriented across transitions between related statistical graphics with research to date primarily focused on transitions in response to filtering, time steps, changing variables, or adjusting visual encodings (Kim et al., 2019).

### Feedback, Interaction, and Error Prevention: Supportiveness and Robustness

The evaluation of the usability of data visualization tools can be carried out by studying the errors made by the user with the objective of introducing improvements for future prevention and for enhancing their *robustness*. The general question is: “How many errors do users make, how severe are these errors, and how easily can they recover from the errors?” (Nielsen Norman Group, 2012). The *supportiveness* is a requirement that seeks to empower the user for his success through training services, help, support consultation generated by self-learning automated systems that identify and correct errors, and irregularities. Applied to data visualization tools, such as Lyra, this ability has been studied in association with their interactive capacity (Satyanarayan and Heer, 2010). Interactive visualizations have been incorporated into the design of applications in the context of machine comprehension based on error analysis, for example, in NLP (natural language processing) such as Errudite (Wu et al., 2019). In order to understand the user’s participation in the content of the visualization, making them part of a social process and a learning community, there are tools developed to help users in order to obtain better visualizations. Mixed-initiative systems such as Voyager have been designed in order to support “faceted browsing of recommended charts chosen according to statistical and perceptual measures” (Wongsuphasawat et al., 2017).

### Global Experience and Satisfaction

The user’s experience with data visualization is summarized in the satisfaction obtained; so the study of the requirements of data visualization culminates with the explanation of the contributing factors. Determining how attractive the design is to use (Nielsen Norman Group, 2012) “can make a difference on whether or not users come back to it” (Chrisdasie, 2020). Satisfaction refers to the “extent to which the user’s physical, cognitive, and emotional responses that result from the use of a system, product, or service meet the user’s needs and expectations” (ISO, 2020). In the evaluation of visual communication, it has been proposed to obtain early feedback on the level of user satisfaction through questionnaires or qualitative interviews, as well as through analytics of the use of visualization and other more sophisticated techniques such as movement analysis eyes when users use visualization (Agrawala et al., 2011). There are studies on usability and the user satisfaction of hardware–software interfacing visualization that have demonstrated the need to develop educational research on the use of display technologies, such as in the field of learning programming (Ali and Derus, 2014). User *satisfaction* is a requirement that has prompted a large number of studies in the scientific literature, some of which have even proposed the development of an “ontology visualization tool, to provide a user-centered interactive solution” for extracting and visualizing Linked Data (Ghorbel et al., 2017). Experimental evidence indicates that research on systems for evaluating the degree of accomplishment of data visualizations is still incipient. In similar terms that can be stated about the certainty of scientific theories, user’s satisfaction cannot be certified, but dissatisfaction can eventually be demonstrated.

## Results

The results of the study conducted in this article can be classified into two groups: *theoretical—*which include (a) dimensional factors, (b) characterization of achievements—and *practical*, which include (c) types of data visualization, (d) functions, (e) principles of assessment, and (f) professional competences of data visualization.

a) DIMENSIONAL TAXONOMY OF DATA VISUALIZATION. Table 2 shows the dimensional taxonomy with indication of the factors of completeness and complexity for each stage of procedure and progress of data visualization.
b) CHARACTERIZATION OF ACHIEVEMENTS. The nature of the conditions or properties in the procedure of data visualization follows a common pattern of a sequential order. Table 3 shows the following: in the basic layer, substantial or essential conditions that must be achieved by data visualization; in the extended, formal conditions; in the synthetic, modal conditions; in the dynamic, objective conditions; in the interactive, demonstrative conditions; and finally in the integrative layer, transcendental conditions. From a practical point of view, the design of a dimensional taxonomy of data visualization may cast fresh light on the types, functions, principles, and required competences for data visualization.
c) TYPES OF VISUALIZATION. Once an object-centered model of data visualization has been defined—as previous exploratory and experimental studies have shown (Cavaller et al., 2020)—six types of data visualization can be obtained (see Table 4).
d) FUNCTIONS OF DATA VISUALIZATION. According to the defined taxonomy, factors, and achievements, the functions of data visualization are the following (see Table 5):

1. The first function of data visualization is to show the relationship among the parameters that describe a phenomenon, a process, a system, or any observable subset of the real world.
2. The second is to represent data in a visual way by a graphical representation.
3. The third function is to communicate, that is, to convey meaning—transforming data into information—to be understood by someone.
4. The fourth function is the dissemination of a meaning content by a graphic design appropriate to the context where it will be communicated.
5. The fifth function is to communicate relevant and objective information in the most efficient way through the appropriate media.
6. The sixth function of data visualization is to observe the restraints, capabilities, and conditions from the users in order to enhance the communication performance.

e) PRINCIPLES FOR THE ASSESSMENT OF DATA VISUALIZATION. Data visualization can be assessed according to six different principles of interests.

1. The principle of analytical interest states that data visualization is right in so far as it keeps scientific rigor, order, and method in the quantitative procedures
2. The principle of functional or pragmatic interest states that data visualization is right in so far as the graphical representation has a practical utility and added value over other communicative forms facilitating their comprehension.
3. The principle of managerial interest states that data visualization is right in so far as it is able to package data-message and graphic representation in a singular configuration that promotes the understanding of a meaningful communication.
4. The principle of interest for efficacy states that data visualization is right in so far as, taking into account the professional, social and cultural context and target; it produces the intended communicative result by a suitable design.
5. The principle of interest for efficiency states that data visualization is right in so far as it achieves the communication goals by the optimal means of communication with maximum benefits and minimal use of resources
6. The principle of appraisal interest states that data visualization is right in so far as it receives a positive assessment from the user in terms of usability and of other factors related to H–M interaction.

f) According to the functions and principles mentioned above, data visualization can be defined as a multidisciplinary field where professionals need a wide range of knowledge specializations and professional competences such as data analysis, data graphic representation, programming, graphic design, media publishing, and human–machine interaction.

**TABLE 2:** Dimensional taxonomy of data visualization: factors of completeness and factors of complexity.

		Factors of completeness					
	Elements	Content	Graphic Representation	Encoding Set-up	Graphic design	Media	User
	**Dimensions**	**Degrees** of abstraction	**Functionalities** of the tools for the graphical representation	**Specifications** of the set-up of the visualization	Approach **modes** and **properties** of visualization	**Levels** of communication efficiency	**Requirements** from the user experience side
	**Layers**						
**Factors of complexity**	**Basic**	Parameters and scales	Descriptive graphs	Formalization and basic setup	Subjective	Edition	Perception
	**Extended**	Indicators and relations	Multivariate or relational graphs	Transformation	Objective	Information architecture	Accomplish-ment of tasks
	**Dynamic**	Variable sets distribution	Dynamic graphs	Integration	Informative	Technology and visual tool	KT performance
	**Synthetic**	Processes and phases	Process info graphs and motion graphics	Modelization of processes	Commercial	Mediality: multimediality	Proficiency
	**Interactive**	Hyper-processes layers and systems	Interactive graphics	Interactive visual analysis	Educational	Media interaction	Feedback and errors
	**Integrative**	Organizations and ecosystems	Scientific graph	Ecosystem modeling	Scientific	*Meta*-mediality	Global experience

**TABLE 3:** Dimensional taxonomy of data visualization: properties or conditions of data visualization.

	Visualization element	Content	Graphical representation	Encoding setup	Graphic design	Media	User
**Achievement**	**Essential**	Congruence	Evidentiality	Accuracy	Communicativity	Reliability	Learnability and flexibility
	**Formal**	Exhaustivity	Proportionality and integrity	Multi-directionality	Adaptability	Navigability	Efficiency
	**Modal**	Consistency	Schematicity	Versatility	Appealingness	Accesibility	Effectiveness and performance
	**Objective**	Cohesive unity	Flow logicality	Self-explanatority	Persuasivity	Mediality	Proficiency and memorability
	**Demonstrative**	Falsibiability	Complex evidentiality	Operatibility and transparency	Dialogicity	Participativity	Supportiveness and robustness
	**Transcendental**	Modeling	Intricacy	Ubiquity	Integrativity	Experiment-ability	Satisfaction

**TABLE 4:** Variables, types of visualization, and graphical representation by goals from the perspective of an object-centered data visualization model (Cavaller et al., 2020).

	Content-variable	Types of data visualization and graphical representation	Object-goal
1	Measurements	Descriptive	Parameters and basic relationships
2	Indicators	Relational	Multivariate relationships
3	Distributions	Multi-relational dynamics	Factors or multi-relationships
4	Flow: vector	Process	Internal logics
5	Network: connector	Hyper-process: system	Architecture
6	Program: code	Ecosystem	Organization

**TABLE 5:** Taxonomy of data visualization: functions, principles, and competences in data visualization.

Visualization element	Content	Graphical representation	Setup	Graphic design	Media	User experience
Component	Message	Form	Encoder	Context	Channel	Decoder
Functions	Show parametrical relations	Represent data in a visual way	Convey meaning	Dissemination of information	Efficient communication	Enhance communication performance
Principles	Analytical	Functional	Managerial	Efficacy	Efficiency	Appraisal
Competences	Data analysis	Data graphic representation	Programming	Data graphic design	Media publishing	Human–machine interaction

## Findings and discussion

The fundamental conceptual findings of the study include the following:

1) The process of data visualization can be viewed from the perspective of communication sciences, which includes six major components: message, form, encoder, context, channel, and decoder.
2) These components correspond to six elements in the context of data visualization: content, graphic representation, encoding setup, graphic design and approach, media, and user.
3) The process of data visualization must integrate complexity as a parameter in its implementation, and it must be ordered according to six layers of complexity: basic, extended, dynamic, synthetic, interactive, and integrative. These layers, obtained by analytical criteria, indicate the degree of the internal complexity of the organized entity or a phenomenon that is represented, and they are defined in order to facilitate the systematic application of object-oriented data visualization.

**FIGURE 7:**
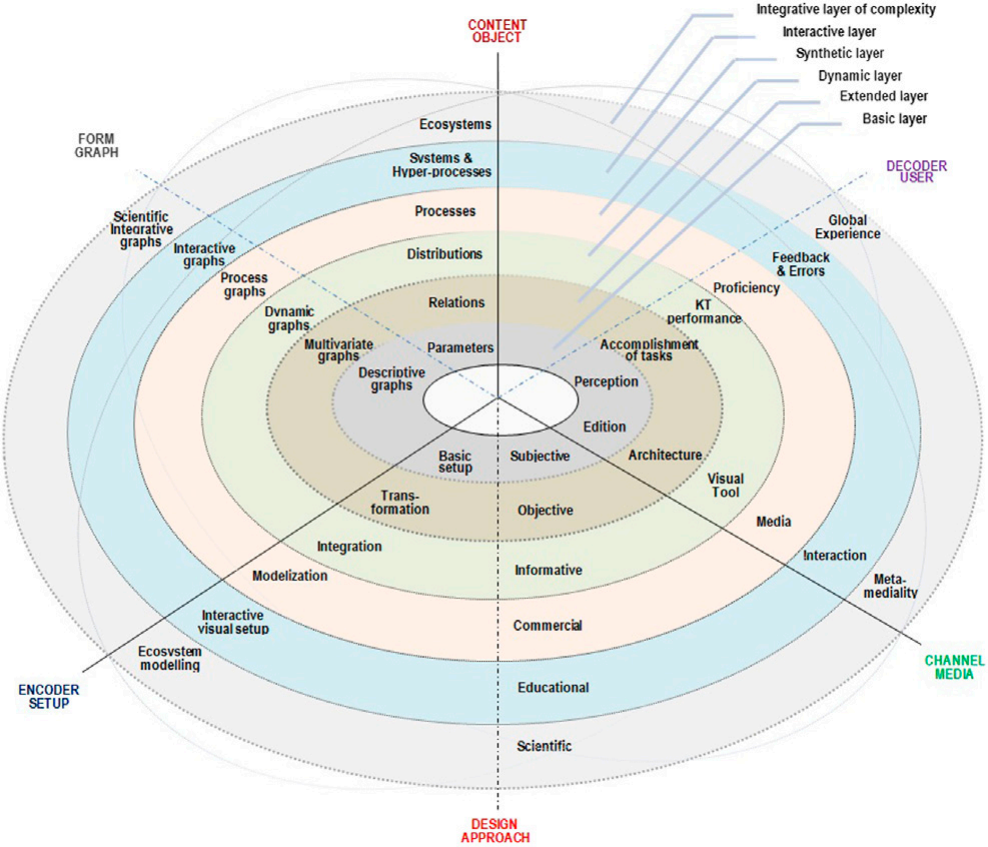
Illustrative representation of the dimensional taxonomy for object-oriented data visualization from the perspective of communication sciences: elements-axes as factors of completeness and layers spheres as factors of complexity. Source: Own elaboration.

The process of data visualization must be addressed following the unfolding of the possibilities that arise from the combination of these factors, reaching the observed achievements at each crossroads between *communication component* x *layer of organizational complexity* (see Figure 7).

Previous theoretic and practical studies have led to the assumption that data visualization is mainly instrumental. Conversely, the results of this study reveal that the potentialities of the analytical functions of data visualization are strictly related to its ability to show the scale and the increasing intricacy of the networked organization of a complex system, in which relationships and processes are interconnected.

In other terms, the efficacy of data visualization not only depends on the completeness of its *extended deployment* taking into account communication factors but also on its *in-depth unfolding* following the level of organizational complexity in which the analysis has been performed. This holistic approach enables data visualization to be understood as the visual representation of knowledge, after data formalization and data analysis. As the key time that culminates and completes data processing, data visualization summarizes the underlying background knowledge that potentially initiates a new inquiry in the innovation cycle.

**FIGURE 8:**
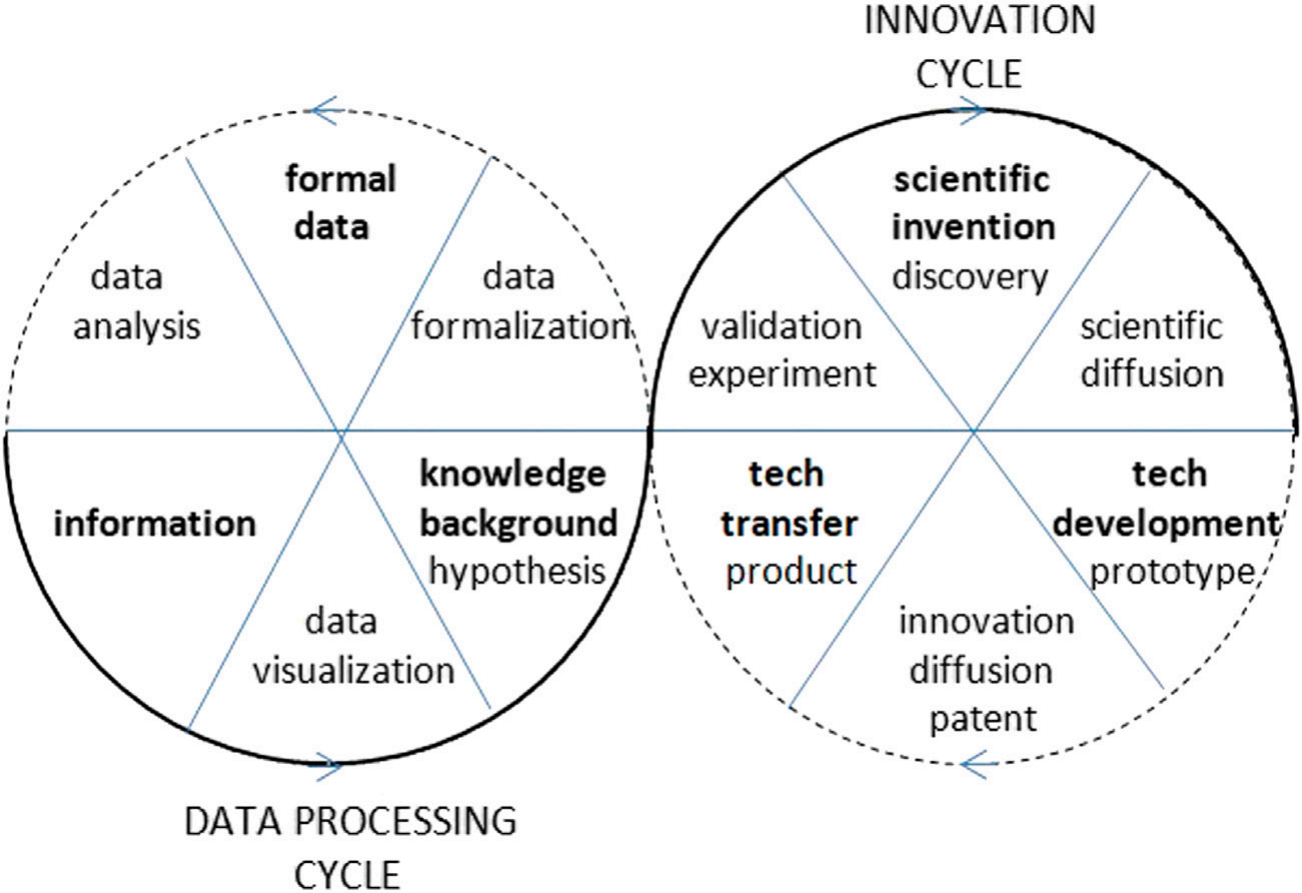
Representation of the sequentiality of data processing and innovation hyper-cycle.

For an open discussion, it must be pointed out that the completion of data visualization, according to the proposed taxonomy, culminates data processing cycle, making visible the knowledge background. On this basis, scientific research, technological development, and transfer deploy the cycle of innovation (Cavaller, 2008), which, in turn, pushes data processing cycle for the extension of scientific knowledge (see Figure 8). So, in a major hyper-cycle, data processing and innovation cycles can be seen as an augmented projection of human cognitive process, where this taxonomy of data visualization can play an extended key role, an issue that constitutes the object for future research actions.

## Data Availability

The original contributions presented in the study are included in the article/Supplementary Material; further inquiries can be directed to the corresponding author.
